# School, family, and peer connectedness as protective factors for depression and suicide risk in Spanish adolescents

**DOI:** 10.3389/fpsyg.2025.1547759

**Published:** 2025-05-22

**Authors:** Yolanda Pastor, Vanesa Pérez-Torres, Ariadna Angulo-Brunet, Juan E. Nebot-Garcia, Elena Gallardo-Nieto

**Affiliations:** ^1^Department of Psychology, Rey Juan Carlos University, Madrid, Spain; ^2^Faculty of Psychology and Educational Sciences, Universitat Oberta de Catalunya, Barcelona, Spain; ^3^Department of Basic and Clinical Psychology and Psychobiology, Universitat Jaume I, Castellón de la Plana, Spain; ^4^Department of Pedagogy, Universitat Rovira I Virgili, Tarragona, Spain

**Keywords:** family connectedness, school connectedness, peer connectedness, depression, suicide, adolescence

## Abstract

**Introduction:**

School, family, and peer connectedness have been shown to be a protective factor for depression and suicide risk in adolescence. However, no comprehensive models have been formulated to assess the influence of each of these factors together on adolescent depression and suicide. The purpose of this study is to analyze the direct and indirect effect—mediated by depressive symptomatology—that different dimensions of social connectedness (family, school, and peers) have on suicide risk.

**Methods:**

A battery of tests on depression, suicide risk, and connectedness was administered to a representative sample of 806 Spanish adolescents aged 14 to 17 (*M* = 16.4, *SD* = 0.74), by means of an online survey through representative panels.

**Results:**

Structural equation models showed that family connectedness reduces the risk of suicide in adolescence, through both its direct and indirect effects, by virtue of the reduction of depressive symptomatology. The other dimensions of connectedness (peer and school), although negatively related to depression and suicide, were not significant predictors in the models.

**Discussion:**

The practical implications of these results argue for the promotion of family connectedness to prevent adolescent suicide and depression. The lack of prediction regarding peer and school connectedness suggests the influence of possible cultural or contextual factors in Spain, making additional research necessary in this regard.

## 1 Introduction

In the scientific literature, relational aspects like connectedness are some of the most important protective factors for mental health and wellbeing (Arango et al., 2019, 2024; Opperman et al., 2015; Resnick et al., 1993). The sense of connection or connectedness stems from what people derive from their relationships with others in the different contexts in which they are involved, or in the words of Townsend and McWhirter (2005), it is produced “when a person is actively involved with another person, object, group, or environment, and that involvement promotes a sense of comfort, wellbeing, and anxiety reduction” (p. 193). Connectedness refers to the sense of belonging, acceptance, respect, safety, feelings of mutual support, engagement, and inclusion in certain contexts (Lee and Robbins, 2000; Too et al., 2022). In the context of mental health prevention in particular, connectedness contributes to active participation in the community. A greater sense of belonging, exposure to positive role models, and a better perception of closeness and support from the environment are among other benefits (Foster et al., 2017).

One of the major concerns in adolescent development research is understanding protective and risk factors for mental health due to the increase in anxiety problems, depression, and suicidal ideation and suicide attempts in this population (Arango et al., 2024; Malaquias et al., 2015). In adolescence, social connectedness can occur in a variety of situations, such as with peers, family, school, and the community. These relationships are all part of the experience of interpersonal closeness with the social world. Adolescent interpersonal connections are a key component of positive development and have been shown to protect against stressors, bringing teenagers stability in the face of social pressures and demands and offering a sense of belonging (Malaquias et al., 2015). Moreover, family, school, and peer connectedness are positively associated with adolescent wellbeing and have been shown to be protective against suicidal behavior and depression (Arango et al., 2019, 2024; Malaquias et al., 2015; Opperman et al., 2015).

With regard to the role of family connectedness, research has shown that healthy adolescent development is influenced by parents through parent-child connectedness, including family cohesion, social support, and specific parenting practices, such as monitoring guidance, respect for individual interest, and open communication (Arango et al., 2019; Borowsky et al., 2001; Malaquias et al., 2015; Yap and Jorm, 2015). Moreover, positive adjustment during adolescence is largely based on positive connectedness in family relationships, which satisfies the needs for relatedness and promotes psychological growth and wellbeing (Yap and Jorm, 2015). Family connectedness refers to the sense of belonging and psychological proximity to family members and feelings of warmth, love, and caring from parents and other family members (Borowsky et al., 2001; Resnick et al., 1993). Research about mental health shows the protective effect of this connection against adolescent emotional discomfort. For example, a longitudinal study conducted with adolescents (Gervais and Jose, 2020) confirmed the beneficial effects of family connectedness, a predictor of better physical, social, and mental health. Moreover, it has been demonstrated that family connectedness is a predictive factor for fewer depressive symptoms among adolescents and youth (Borowsky et al., 2001; Eugene, 2021) and prevents suicidal ideation and behavior (Arango et al., 2019, 2024; Borowsky et al., 2001; Conner et al., 2016; Foster et al., 2017). All of the above suggests the hypothesis that family connectedness reduces suicidal risk among adolescents, with depression being a variable that mediates this relationship. Indeed, a prior review identified the family as the most relevant context for cultivating connectedness for the prevention of adolescent suicide, followed by school, peers, and community (Whitlock et al., 2014). This, together with the preponderant role of familism—understood as the high value of the family unit in terms of respect, support, obligation, and reference (Kapke et al., 2017; Stein et al., 2013; Sánchez-Vera and Bote-Díaz, 2009; Verd et al., 2024)—in the cultures of southern Europe, like Spain and Italy, and its relationship to wellbeing and mental health (Valdivieso-Mora et al., 2016), is consistent with the hypothesis that family connectedness carries greater weight as a protective factor for suicidal risk and depression in adolescence than school or peer connectedness.

Even though the family remains an important social context in adolescents’ lives, there is also considerable evidence about the significance and influence of peers during this period, especially regarding social interactions, social support, and attachment (Foster et al., 2017; Gowing, 2019). Peer connectedness is a term that refers to the perception of support, genuine caring, and trust in one’s peer group (Bernat and Resnick, 2009) and influences mental health during adolescence. For example, depression and suicide have been found to be related to the quality of peer relationships (Prinstein et al., 2000; Whitlock et al., 2014). The protective effect of peers depends on the nature of the social interrelationship. Positive peer relationships promote wellbeing, while rejecting, bullying, or socially isolating behavior from peers can increase psychological distress (Foster et al., 2017; Gowing, 2019). Although a review of the literature suggests that, in general, peer connectedness may provide protection against suicidal thoughts and behaviors, in some cases when a friend attempts suicide or has positive attitudes toward suicide, this may also constitute a risk factor (Whitlock et al., 2014).

Moreover, school is also a relevant social environment during adolescence and is a key component of social connectedness. Adolescent perceptions of school connectedness (e.g., the quality of teacher-student relationships, the school environment, inclusion, feelings of belonging, acceptance, and interpersonal support) are related to positive school adjustments, improved academic achievement, overall health, and mental wellbeing (Bersamin et al., 2019; Gowing, 2019). Participating in school produces a sense of wellbeing and a decrease in depressive symptoms among young people (Borowsky et al., 2001). The learning environment and a positive relationship with one’s school are other factors that contribute to wellbeing in adolescence. Connectedness to the school is related to lower symptoms of depression and better academic achievement, and enhances self-efficacy (Bersamin et al., 2019; Gowing, 2019; Malaquias et al., 2015; Raniti et al., 2022). A longitudinal study conducted in Australia found that students with higher school and social connectedness had a lower risk of anxiety and depressive symptoms over time (Bond et al., 2007). Furthermore, with young people who experienced electronic victimization, higher school connectedness was prospectively linked to less suicidal behavior (Kim et al., 2020). At the longitudinal level, it has been found that higher school connectedness was associated with a lower probability of suicide attempts over 6 months (Arango et al., 2024). A recent systematic review also showed that school connectedness is associated with less suicidal ideation in adolescence in most of the literature (73.3%) and fewer suicide attempts in half of the studies reviewed (50%), although the authors underscore the importance of examining the potential moderators of this relationship (Welty et al., 2024). Another meta-analytic review also reported the negative relationship of school connectedness with both suicidal ideation and suicide attempts (Marraccini and Brier, 2017).

This study tests two complementary models of the relationship that family, school, and peer connectedness have on adolescent suicide risk (model 2) and depression (model 3), after testing the measurement model (model 1). Model 2 assesses the possible influence of connectedness in different environments (school, family, and peers) on suicide risk. Model 3 starts from the variables that showed a relevant relationship in model 2, to examine the mediating role of depressive feelings in explaining suicide risk. The testing of comprehensive models makes it possible to assess the greater or lesser influence that each of these agents (school, family, and peers) has on depression and the risk of suicide in adolescence.

The hypotheses tested with model 2 are: The greater the family connectedness (hypothesis 1), the higher the school connectedness (hypothesis 2) and the greater the peer connectedness (hypothesis 3), the lower the suicide risk will be in adolescence. The model 3 hypotheses are: Depressive feelings positively influence suicide risk in adolescence (hypothesis 4), and the greater the family connectedness (hypothesis 5), the higher the school connectedness (hypothesis 6), and the greater the peer connectedness (hypothesis 7), the lower the depressive feelings will be in adolescence. Family connectedness also has a direct negative relationship with suicide risk in adolescence (hypothesis 8).

## 2 Materials and methods

### 2.1 Participants

A nationally representative sample of 806 Spanish adolescents between 14 and 17 years of age was obtained (*M* = 16.4, *SD* = 0.74, Median = 16). Of them, 46.8% (*n* = 377) were cis women, 49.6% (*n* = 400) were cis men, 0.6% (*n* = 5) were trans women, 0.9% (*n* = 7) were trans men, 1.4% were non-binary (*n* = 11), and 0.8% (*n* = 6) were unsure.

Most of the participants were born in Spain (86.2%; *n* = 695) and the majority had Spanish nationality (86.1%, *n* = 694). As for their ethnic background, 69.6% (*n* = 561) described themselves as White or Caucasian, 16.6% (*n* = 134) as Hispanic, 4.8% (*n* = 39) as Arabic or Maghrebi, 4.6% (*n* = 37) as Roma, 3.3% (*n* = 27) as being of African extraction, and 1% (*n* = 8) as Asian. With regard to the type of school, 69.4% (*n* = 144) of the participants were attending public schools, 17.9% (*n* = 144) charter schools, and 12.8% (*n* = 103) private schools. 41.4% (*n* = 334) of the schools had no religious affiliation.

### 2.2 Measures

#### 2.2.1 Connectedness

To assess school, family, and peer connectedness, we used an adaptation of these subscales to Spanish from the Self in a Social Context-Social Connectedness Scale (Carroll et al., 2017). This scale has 35 items: peer connectedness (15), school connectedness (9), and family connectedness (11). This is a 4-point Likert scale (1 = “not at all” to 4 = “all of the time”), all the items are direct, and a high score expresses a high connectedness in each dimension. In this sample, we obtained positive evidence of internal structure validity through a 3-factor correlated model [X^2^(*df*) = 1622.5 (557), CFI = 0.97, TLI = 0.97, RMSEA (90% CI) = 0.05 (0.05, 0.05)]. Internal consistency reliability was excellent for the three dimensions: peer (α = 0.94, *ω_*c*_* = 0.96), family (α = 0.94, *ω_*c*_* = 0.95), and school (α = 0.91, *ω_*c*_* = 0.93).

#### 2.2.2 Depression

We used the Spanish adaptation (Fonseca-Pedrero et al., 2023; González-Blanch et al., 2018) of the Patient Health Questionnaire-9 (PHQ-9; Burdzovic and Brunborg, 2017) for adolescents. This has nine items with a 4-point Likert scale (0 = “never” to 3 = “almost every day”). All the items are direct and a high score represents a high level of depressive symptomatology. In this sample, we obtained positive evidence for a one-factor model [*X*^2^(*df*) = 172.6 (27), CFI = 0.98, TLI = 0.97, RMSEA (90% CI) = 0.08 (0.07, 0.09)] and adequate internal consistency reliability (**A** = 0.89, Ω*_*c*_* = 0.90).

#### 2.2.3 Suicidal risk

We used a Spanish-language version of the Paykel Suicide Scale (Fonseca-Pedrero and de Albéniz, 2020). This is a 5-item, dichotomous scale (0 = “No”; 1 = “Yes”), and a high score indicates a greater risk of suicide. The questionnaire evaluates suicidal ideation and past behavior. We had excellent goodness of fit indexes (GOFI) for a one-factor model in this sample [*X*^2^(*df*) = 15.7 (5), CFI = 1.00, TLI = 1.00, RMSEA (90% CI) = 0.05 (0.02, 0.08)]. Internal consistency reliability was adequate for research purposes (α = 0.83, *ω_*c*_* = 0.85).

### 2.3 Data analysis

The recommendations of Doval et al. (2023) were used to choose the SEM estimators and the internal consistency reliability coefficients. Given that the data were ordinal (4-point Likert scale) or dichotomous (true/false) and no missing data were present, the weighted least squares mean and variance corrected estimator (WLSMV) was used and the categorical omega (*ω_*c*_)* and Cronbach’s alpha (α) were provided for each scale in the Measures section. To assess the GOFI of the models, values greater than 0.95 in CFI and TLI and less than 0.05 in RMSEA were considered excellent (Hu and Bentler, 1999; Xia and Yang, 2019), and values greater than 0.90 in CFI and TLI and less than 0.08 in RMSEA adequate (Marsh et al., 2004).

To test the structural equation models (SEMs), we first tested the measurement model (model 1) including all the measures with the aforementioned estimators that are optimal when data are categorical (see Savalei, 2014 for a more detailed explanation). We then fit a second model (model 2) in which the three connectedness dimensions were predicting suicidal behavior in order to assess hypotheses 1–3. Finally, a third model (model 3) was tested in which connectedness to family, school, and peers was related to depression, and family connectedness directly and indirectly—mediated by depression—influenced suicide risk (hypotheses 4–8). As no previous direct effect of school and peer connectedness with suicide risk was found, we decided not to include these relationships in Model 3.

All the analyses were performed using R software (R Core Team, 2024; version 4.4.1) and the SEM was performed using lavaan (Rosseel, 2012). We followed Caughlin (2022) recommendations for performing and interpreting the meditation results. In that process, 5,000 bootstrapping procedures were performed using DWLS to obtain 95% confidence intervals (CI) for the indirect effect. The CI reported in this work were all obtained by bootstrapping.

In order to interpret the strength of the correlations, we followed Cohen (1988) criteria (<0.30 low, between < 0.30 and 0.50 moderate, > 0.50 high).

### 2.4 Procedure

A nationally representative sample stratified by region and sex assigned at birth of Spanish adolescents aged 14–17 years was collected using online panels. A total of 2,984 individuals started the survey, and 806 completed the survey, with no missing data, because the platform collected participants until we obtained the number of completed surveys required for the study. This corresponds to a response rate of 27%. This sampling considered a confidence interval of 95% and a sampling error of ± 3.45. To conduct the study, the measurement instruments were first adapted to Spanish. For this, a standardized back-translation procedure was used (Bullinger et al., 1993; Callegaro-Borsa et al., 2012), involving two independent bilingual translators, both experts in psychology. The members of the research team compared the different versions, evaluating their semantic, idiomatic, comprehensive, and conceptual equivalence, and suggesting appropriate modifications to ensure equivalence with the original instrument. In addition, the items were adjusted to colloquial language and expressions to ensure their relevance to Spanish adolescents. The questionnaires were administered using a computer-assisted web survey system by a sampling company; informed consents had previously been requested. The study used a cross-sectional design, and the procedure was previously approved by the research ethics committee at our institution.

## 3 Results

Table 1 presents the descriptive statistics and correlations between the main variables of the study, calculated based on the sum or average of items. As can be observed, the three connectedness variables have a moderate-high mean, with family connectedness having the highest score and school connectedness the lowest.

**TABLE 1:** Mean, standard deviation, and Pearson’s correlation of the main study variables.

Characteristic	*M* (*SD*)	1	2	3	4	5
(1) Connectedness with friends	3.07 (0.68)	1.00				
(2) Connectedness with family	3.17 (0.76)	0.52*	1.00			
(3) Connectedness with school	2.88 (0.74)	0.50*	0.51*	1.00		
(4) Depression^a^	9.00 (7.00)	−0.22*	−0.32*	−0.21*	1.00	
(5) Suicidal risk^a^	1.32 (1.67)	−0.16	−0.35*	−0.23*	0.51*	1.00

*p < 0.05; This table provides unstandardized statistics.

^a^The mean of the total sum scores.

On the other hand, the participants, on average, show low scores for depression and suicidal ideation. The correlation between connectedness and depression and suicidal ideation ranges from low to moderate. The negative correlation between family connectedness and depression and suicidal risk is notable.

**FIGURE 1:**
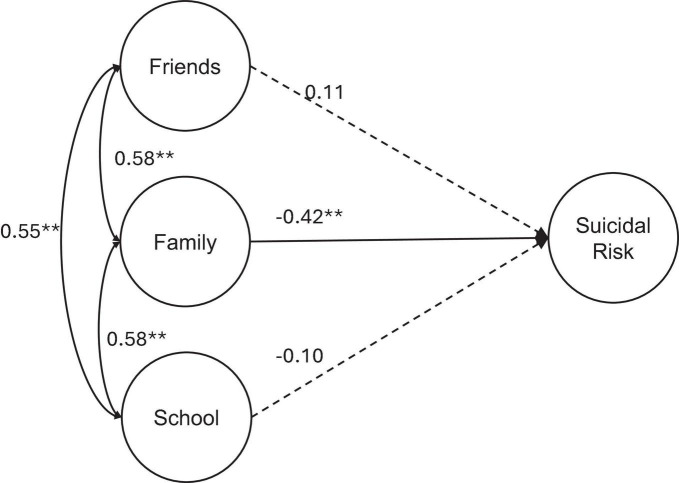
Structural equation model 2: predicting suicidal risk in adolescents. Direct Model. **p < 0.001.

Table 2 presents the GOFI of the tested models. Model 1 includes the measurement model incorporating the three dimensions of connectedness, depression, and suicidal risk. As seen in the table, the GOFI are satisfactory. Model 2 (Figure 1 and Table 3) includes the parameters for the direct model between the three dimensions of connectedness and suicide risk. The measurement model of the depression variable was excluded from Model 2. As shown in Figure 1 and Table 3, there is an inverse relationship between family connectedness and suicidal risk (B = −0.42, *p* < 0.001). There is no effect for friends or school connectedness. For that reason, we did not take the mediation between these variables and depression into consideration.

**TABLE 2:** Goodness of fit indexes for the models.

Model	*X*^2^ (*df*)	*p*	CFI	TLI	RMSEA (90% IC)
Model 1. Measurement model	2,182.41 (1,117)	<0.001	0.975	0.973	0.03 (0.04, 0.04)
Model 2. Direct model	1,713.36 (734)	<0.001	0.973	0.971	0.04 (0.04, 0.04)
Model 3. Mediational model (only family)	2,168.56 (1,119)	<0.001	0.975	0.974	0.03 (0.03, 0.04)

**TABLE 3:** Unstandardized parameters for the models.

Model/parameter	*B*	95% CI	SE	*Z*	*p*	β
**Model 2 (*R*^2^ = 0.19)**						
**Suicidal risk**						
Friends	0.11	−0.02, 0.23	0.06	1.72	0.09	0.10
Family	−0.42	−0.54, −0.30	0.06	−7.00	<0.001	−0.43
School	−0.10	−0.22, 0.03	0.06	−1.50	0.13	−0.09
**Model 3**						
**Depression (*R*^2^ = 0.15)**						
Friends	−0.03	−0.13, 0.08	0.05	−0.55	0.58	0.03
Family (*a*)	−0.31	−0.41, −0.22	0.04	−6.98	<0.001	−0.35
School	−0.04	−0.15, 0.08	0.05	−0.73	0.46	−0.04
**Suicidal risk (*R*^2^ = 0.44)**						
Depression (b)	0.62	0.52, 0.72	0.05	12.17	<0.001	0.57
Family (c)	−0.19	−0.28, −0.10	0.05	−4.04	<0.001	−0.19
Family × depression (a × b)	−0.19	−0.26, −0.13	0.03	−6.12	<0.001	−0.20
Total effect [c + (a × b)]	−0.38	−0.47, −0.28	0.05	−8.09	<0.001	−0.39

95% CI was obtained by bootstrap with 5,000 resamples.

**FIGURE 2:**
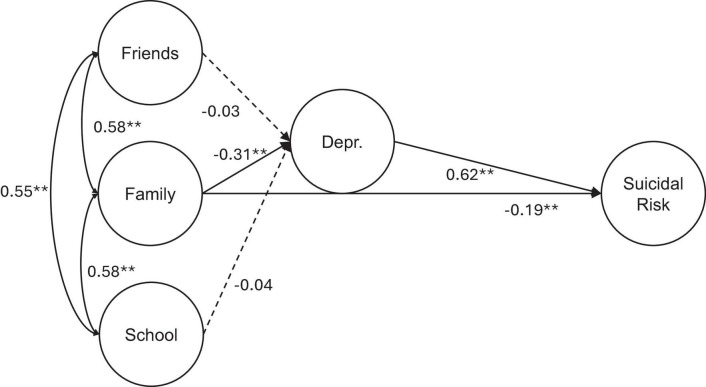
Structural equation model 3: family connectedness and suicidal risk mediated by depression. **p < 0.001.

Finally, Model 3 proposes depression as a mediator between connectedness with family and suicide risk, and school and peer connectedness as related to depression. As seen in Figure 2, there is a partial mediation by depression. Compared with Model 2, the direct effect of family connectedness on suicidal risk has decreased but has not disappeared. The indirect effect is significant (*B* = −0.19 CI 95% −0.26, −0.13).

## 4 Discussion

This paper tests two complementary models designed to assess the role of connectedness (family, school, and peers) as a protective factor for depression and suicide risk among adolescents using a representative sample of Spanish teenagers. Our results supported the measurement model (M1), which includes questionnaires on depression (PHQ-9) (Burdzovic and Brunborg, 2017; Fonseca-Pedrero et al., 2023; González-Blanch et al., 2018), suicidal risk (Paykel Suicide scale; Fonseca-Pedrero and de Albéniz, 2020), and school, peer, and family connectedness (subscales of the Self in a Social Context-Social Connectedness Scale; Carroll et al., 2017). This guaranteed the goodness of fit of the instruments used for these variables.

Regarding the protective role of school, family, and peer connectedness on suicide risk (M2), our model showed that only family connectedness is a protective factor for suicide risk among Spanish adolescents, thus confirming hypothesis 1, but rejecting hypotheses 2 and 3. Previous research supports the protective role of family connectedness in the face of suicide (Arango et al., 2019, 2024; Conner et al., 2016; Foster et al., 2017). This result highlights the importance of the family as a fundamental relational environment to promote wellbeing and protect against suicide. In a review study that compared the results of connectedness in different contexts, family connectedness also emerged as the strongest factor in the prevention of adolescent suicide, ahead of school and peer relationships (Whitlock et al., 2014). The values of Spanish culture regarding the importance of the family (familism) and its relationship with mental health may also explain this result (Kapke et al., 2017; Valdivieso-Mora et al., 2016; Verd et al., 2024). In fact, compared to other European countries, young Spaniards spend more time with their families, maintain daily contact with their parents, and feel more affective proximity to their families (Verd et al., 2024).

With respect to school connectedness, a recent systematic review study identified it as a protective factor for suicidal ideation in 74.4% of the studies, and for suicidal behavior in 50% (Welty et al., 2024). The absence of such relationship in our study may be due to several factors. Many studies that analyze school connectedness do not test comprehensive models that also incorporate family connectedness. As discussed above, family connectedness emerged as the strongest predictor of suicide risk in a review study (Whitlock et al., 2014). Furthermore, comparing Spanish educational policy with that of other European countries, such as Finland or Sweden, the incorporation of mental health and wellbeing as an educational objective is recent, and in the development process. The increasing use of technology, high rates of bullying, the recent incorporation of wellness policies (that are not accompanied by funding or specific training to implement comprehensive preventive measures for mental health and, therefore, do not adequately prepare teachers to meet the new challenges of adolescence) can produce schools that do not constitute a safe and warm environment for teens in Spain. Indeed, the lower mean score observed on school connectedness in our data may well point in this direction. It should also be noted that Spain does not have a comprehensive program to promote mental health in schools. Each institution adopts the measures it deems appropriate. Further research is needed to address the factors that influence school connectedness in the Spanish context.

As with our data, the variable related to connectedness with peers did not appear to be related to suicide in previous studies with socially vulnerable adolescents either (Foster et al., 2017), or in adolescents who had experienced victimization in a longitudinal study (Arango et al., 2024). One possible explanation for this lack of association may be that during this stage, relationships with peers are under construction, involving complex processes, and therefore that experiences of connection are accompanied by experiences of peer rejection and the selection of friends (Laursen and Veenstra, 2021). Another potential explanation, which needs to be verified, is the possible deterioration of connectedness with peers due to the increased use of social media to the detriment of face-to-face relationships, which provide a greater sense of support and connection (Boer et al., 2021). In this respect, further research is necessary to assess how the use of social media is transforming and contributing to peer relationships.

The results shown in Model 3 suggest that neither peer nor school connectedness maintained a significant relationship with depression—refuting hypotheses 6 and 7—and that, once again, connectedness with family appears as a protector of depression and suicide risk, both directly and indirectly (through mediation with depression), thus confirming hypotheses 4, 5, and 8. Previous studies support this strong relationship between family ties (connectedness) and adolescent depression and suicide (Arango et al., 2019, 2024; Borowsky et al., 2001; Conner et al., 2016; Eugene, 2021; Foster et al., 2017). Even though adolescents often look to their peers, care about and aim to enhance these relationships (Laursen and Veenstra, 2021), it is the family environment that protects them from depression and suicide. Family relationships are the basis for building other connections that provide teenagers with security and a sense of wellbeing. Importantly, our data suggest that family connectedness not only reduces the risk of suicide by diminishing the experience of depression in adolescence, but also directly protects against suicide. This may be because there are other possible mediating variables between family connectedness and suicide risk. For example, adolescents who enjoy positive family relationships, characterized by high support and cohesion, show higher academic achievement, self-concept, social skills, and self-esteem, and have fewer socioemotional difficulties and more resilience (Foster et al., 2017; Murillo-Casas et al., 2015; Preston et al., 2016; Tian et al., 2018). When adolescents become involved in negative situations, such as school victimization, high connectedness in the family can act as a protective factor to cope more adaptively with this experience (Duggins et al., 2016). Family connectedness also facilitates the learning of adaptive coping strategies in adolescence, which in turn influences a lower experience of stress (Gervais and Jose, 2024). In addition to this effect, mediated by other variables, family connectedness can also have a direct protective effect on adolescent suicide risk. Consistent with this idea, social support theorists have argued that this variable has both a direct and indirect effect on psychological wellbeing (Gençöz et al., 2004).

## 5 Limitations and future directions

The study presented here is not without limitations. These include the cross-sectional nature of the research design, which precludes conclusions about causality. The use of representative panels to administer the questionnaires—although a very commonly used tool in current research—may result in a lack of representation among adolescents (e.g., individuals from the upper classes or lower socioeconomic backgrounds). In particular, the youngest adolescents in our sample, aged 14 and 15, are less represented in the panels than those aged 16 and 17. In this respect, a weighting factor in the analyses was used to ensure the representativeness of the data.

Our study suggests future lines for research, such as exploring the role of other variables that mediate the relationship between family connectedness and suicide risk during adolescence and investigating why school and peer connectedness do not seem to be such a powerful protector of depression and suicide in Spanish adolescents, whether this is due to contextual, cultural, or other factors. The practical implications of this study indicate the advisability of fostering connectedness in different environments (family, school, and peers, since they show a negative correlation with depression and risk of suicide), paying particular attention to fostering family relationships, which are the basis of wellbeing and serve as a protective factor for both depression and suicide among Spanish adolescents. The implementation of family connectedness focused programs is the most powerful strategy to prevent adolescent depression and suicidal risk among the Spanish population. Providing families with resources and support to provide healthy parenting in the adolescent stage is fundamental. To that end, it is necessary to develop policies and communities that encourage and support family life. Currently, Spanish policies aimed at family reconciliation and adapting the working day protect children, but not adolescents, with these benefits being reduced when children reach the age of 8 and eliminated when they reach 12. This results in longer working hours for parents and less family time during adolescence. All of this, coupled with the generation gap, the development of necessary autonomy in adolescence, and the spread of social media use can lead to greater disconnection in the family. Therefore, it is important to foster communication and parenting skills and to raise awareness about the importance of sharing quality time with adolescent sons and daughters, just as it is necessary to develop policies and strengthen communities to support family life. Finally, education professionals must be provided with resources and training to handle the challenges of adolescence today, and national plans must be implemented to promote wellness and mental health in schools.

## Data Availability

The raw data supporting the conclusions of this article will be made available by the authors, without undue reservation.
